# Supplementary material for: Structure of a monomeric photosystem I core associated with iron-stress-induced-A proteins from Anabaena sp. PCC 7120
Source: Nat Commun. 2023 Feb 17;14:920. doi: 10.1038/s41467-023-36504-1 (PMC9938196; doi:10.1038/s41467-023-36504-1)
Supplement: Supplementary file 1 — Supplementary Information [file 41467_2023_36504_MOESM1_ESM.pdf]

Supplementary Information

Structure of a monomeric photosystem I core associated with iron-stress-induced-A proteins from *Anabaena* sp. PCC 7120

Ryo Nagao[1,8,†,*], Koji Kato[1,9,†], Tasuku Hamaguchi[2,10,†], Yoshifumi Ueno[3,11], Naoki Tsuboshita[1], Shota Shimizu[1], Miyu Furutani[3], Shigeki Ehira[4], Yoshiki Nakajima[1], Keisuke Kawakami[2], Takehiro Suzuki[5], Naoshi Dohmae[5], Seiji Akimoto[3,*], Koji Yonekura[2,6,7,*], and Jian-Ren Shen[1,*]

[1]Research Institute for Interdisciplinary Science and Graduate School of Natural Science and Technology, Okayama University, Okayama 700-8530, Japan
[2]Biostructural Mechanism Laboratory, RIKEN SPring-8 Center, Hyogo 679-5148, Japan.
[3]Graduate School of Science, Kobe University, Hyogo 657-8501, Japan
[4]Department of Biological Sciences, Graduate School of Science, Tokyo Metropolitan University, Tokyo 192-0397, Japan
[5]Biomolecular Characterization Unit, RIKEN Center for Sustainable Resource Science, Saitama 351-0198, Japan
[6]Institute of Multidisciplinary Research for Advanced Materials, Tohoku University, Miyagi 980-8577, Japan
[7]Advanced Electron Microscope Development Unit, RIKEN-JEOL Collaboration Center, RIKEN Baton Zone Program, Hyogo 679-5148, Japan

Present address:
[8]Faculty of Agriculture, Shizuoka University, Shizuoka 422-8529, Japan
[9]Structural Biology Division, Japan Synchrotron Radiation Research Institute (JASRI), Hyogo 679-5198, Japan
[10]Institute of Multidisciplinary Research for Advanced Materials, Tohoku University, Miyagi 980-8577, Japan
[11]Institute of Arts and Science, Tokyo University of Science, Tokyo 162-8601, Japan

[†]These authors contributed equally to this work.

*Corresponding Authors:
Ryo Nagao, E-mail: nagao.ryo@shizuoka.ac.jp
Seiji Akimoto, E-mail: akimoto@hawk.kobe-u.ac.jp
Koji Yonekura, E-mail: yone@spring8.or.jp
Jian-Ren Shen, E-mail: shen@cc.okayama-u.ac.jp

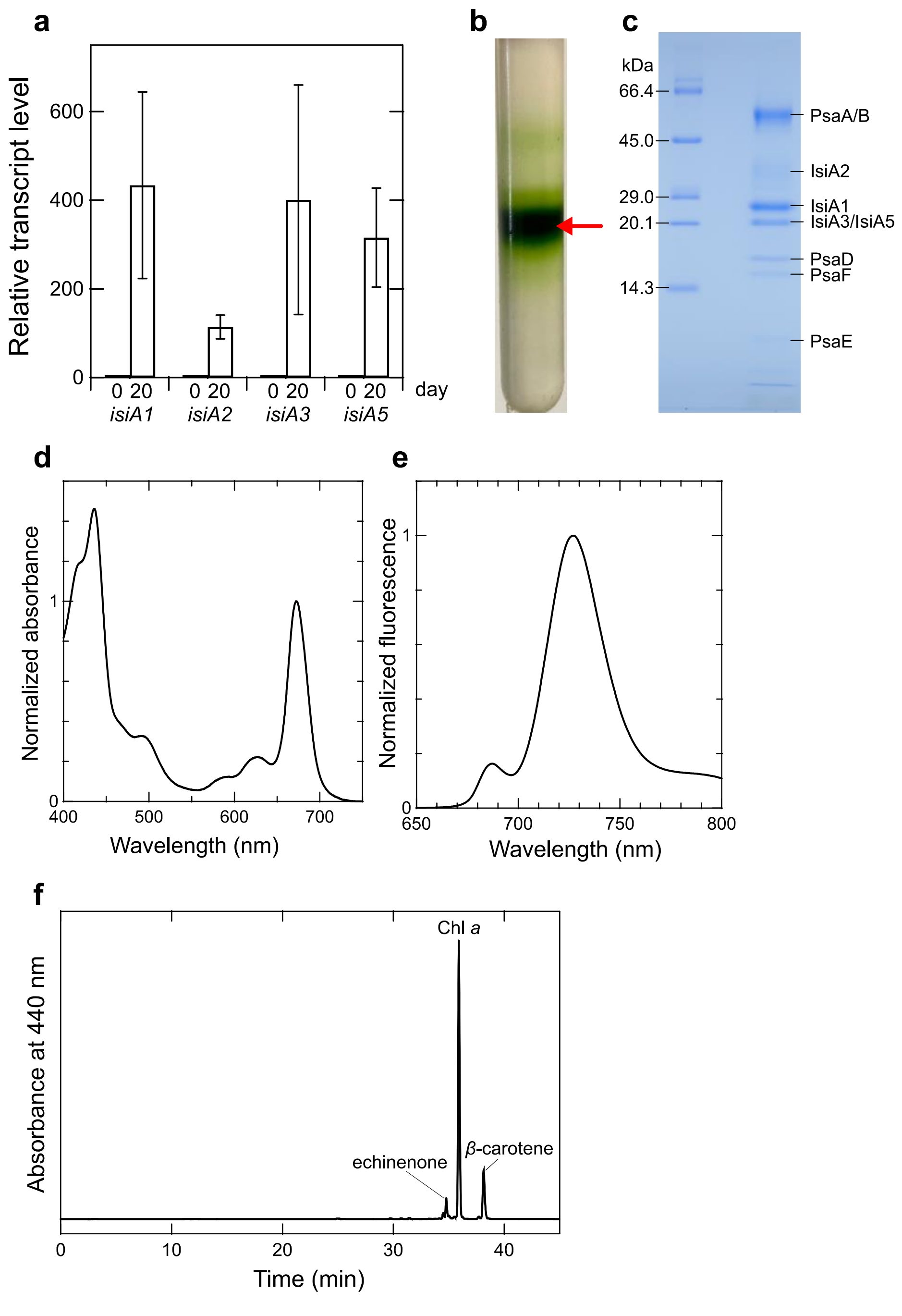

Supplementary Fig. 1. Expression of the *isiA* genes, and purification, characterization of the *Anabaena* PSI-IsiA supercomplex.

a, Expression of the *isiA* genes. Transcript levels of *isiA1*, *isiA2*, *isiA3*, and *isiA5* were determined by qRT-PCR for cells grown under the iron-deficient condition for 0 or 20 days. The transcript level for each gene before iron deficiency (0 day) was taken as 1. RNA samples were prepared from four independently grown cultures, and bars and error bars represent means ± S.D. (n = 4). **b,** Trehalose density gradient centrifugation. The red arrow indicates the PSI-IsiA fraction. **c,** SDS-PAGE analysis of PSI-IsiA. Each band was assigned by mass spectrometry analysis. **d,**

Absorption spectrum of PSI-IsiA measured under room-temperature conditions. Three measurements were averaged, and the resultant spectrum was normalized by the intensity of the Qy peak. **e,** Fluorescence-emission spectrum of PSI-IsiA measured at 77 K upon excitation at 430 nm. Three measurements were averaged, and the resultant spectrum was normalized by the intensity of the PSI fluorescence at 727 nm. **f,** HPLC analysis of the pigments extracted from PSI-IsiA monitored at 440 nm. Data in panels **b, c, f,** are representative of three independent experiments. Source data are provided as a Source Data file.

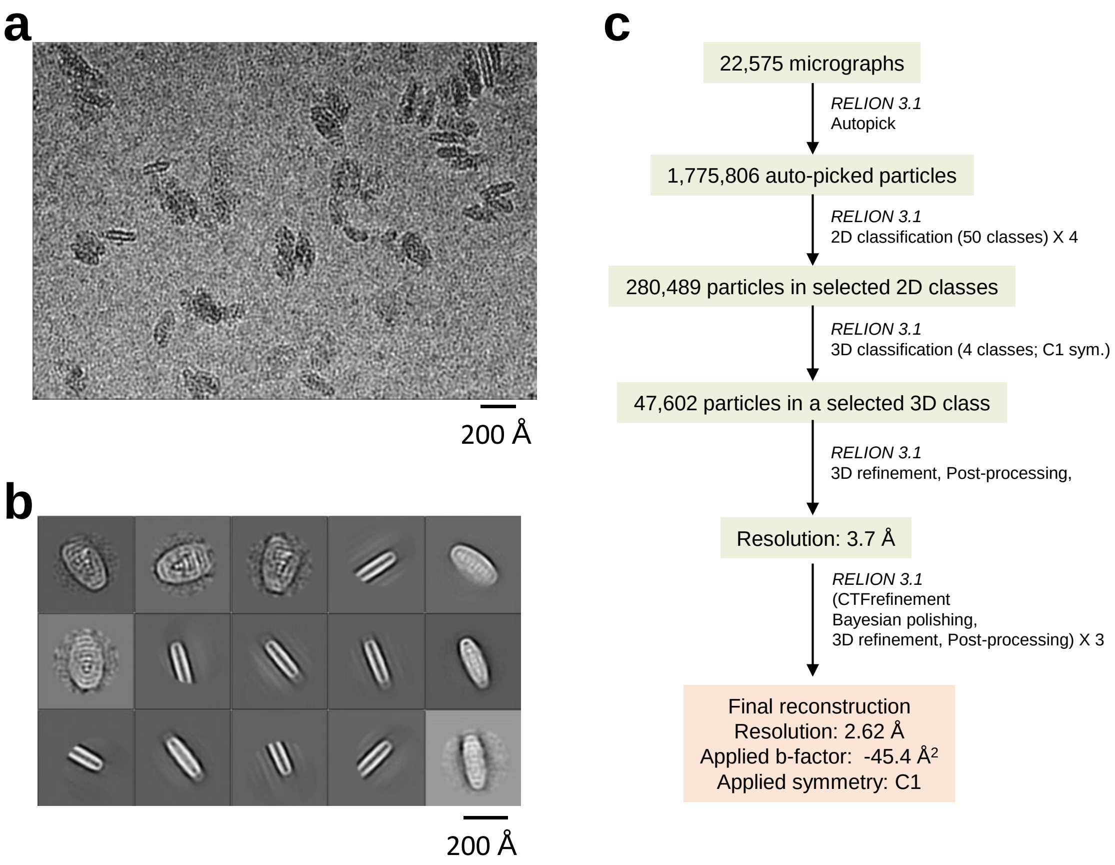

Supplementary Fig. 2. Cryo-EM data collection and processing of PSI-IsiA.

a, A representative cryo-EM micrograph of PSI-IsiA in 22,575 micrographs. **b,** Representative 2D classes of PSI-IsiA. The box size is 396 Å. **c,** Schematic flowchart showing the classification scheme and data processing for PSI-IsiA. The overall PSI-IsiA structure was reconstructed at 2.62-Å resolution from 47,602 particles. See Methods section for more details.

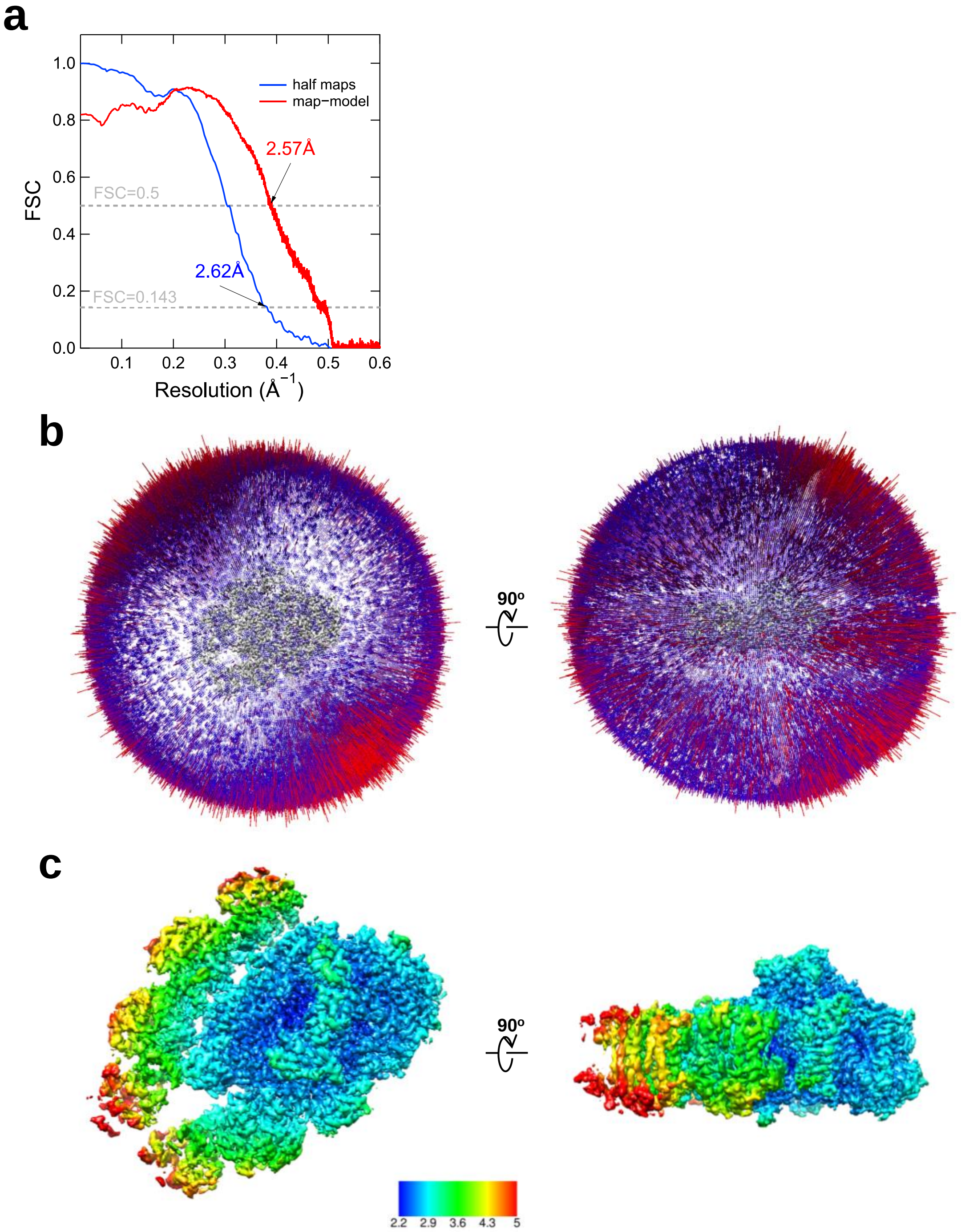

Supplementary Fig. 3. Evaluation of the cryo-EM map quality.

a, FSC curves of PSI-IsiA for independently refined half maps (blue) and map-minus-model (red). **b,** Angular distributions of the particles used for the reconstruction of PSI-IsiA. Each cylinder represents one view, and the height of the cylinder is proportional to the number of particles for that view. **c,** Local resolution maps of PSI-IsiA.

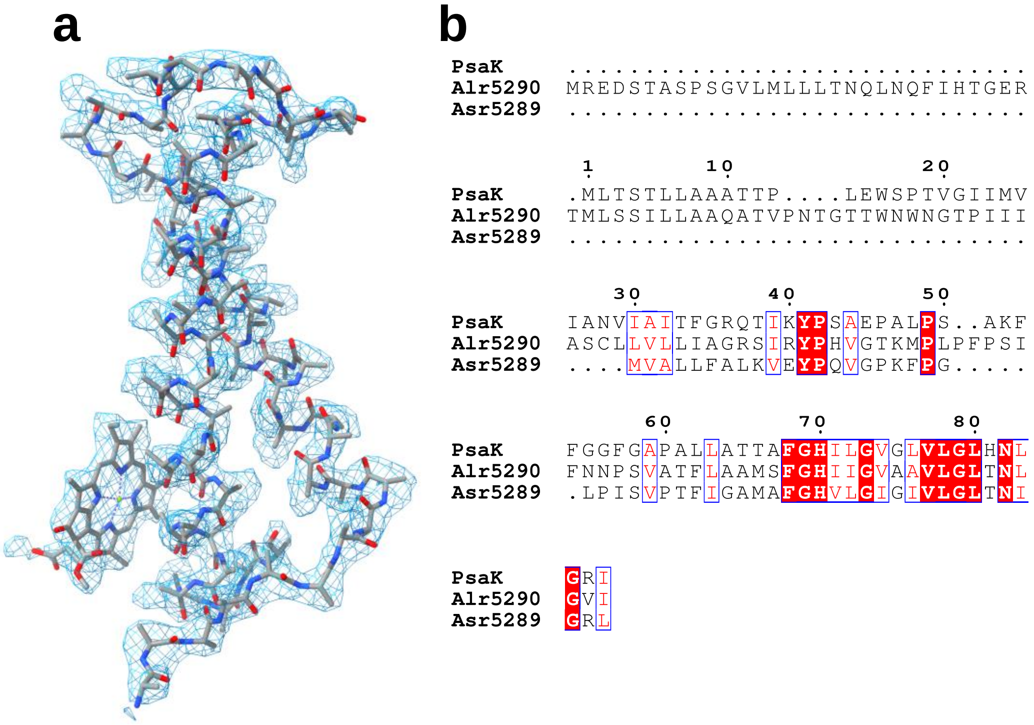

Supplementary Fig. 4. Evaluation of the structure at the position of PsaK.

a, The density for Unknown and its corresponding polyalanine model are shown as blue meshes and gray sticks, respectively. **b,** Multiple sequence alignment (ClustalW and ESPript) among three PsaK family proteins (PsaK, Alr5290, Asr5289) in *Anabaena*.

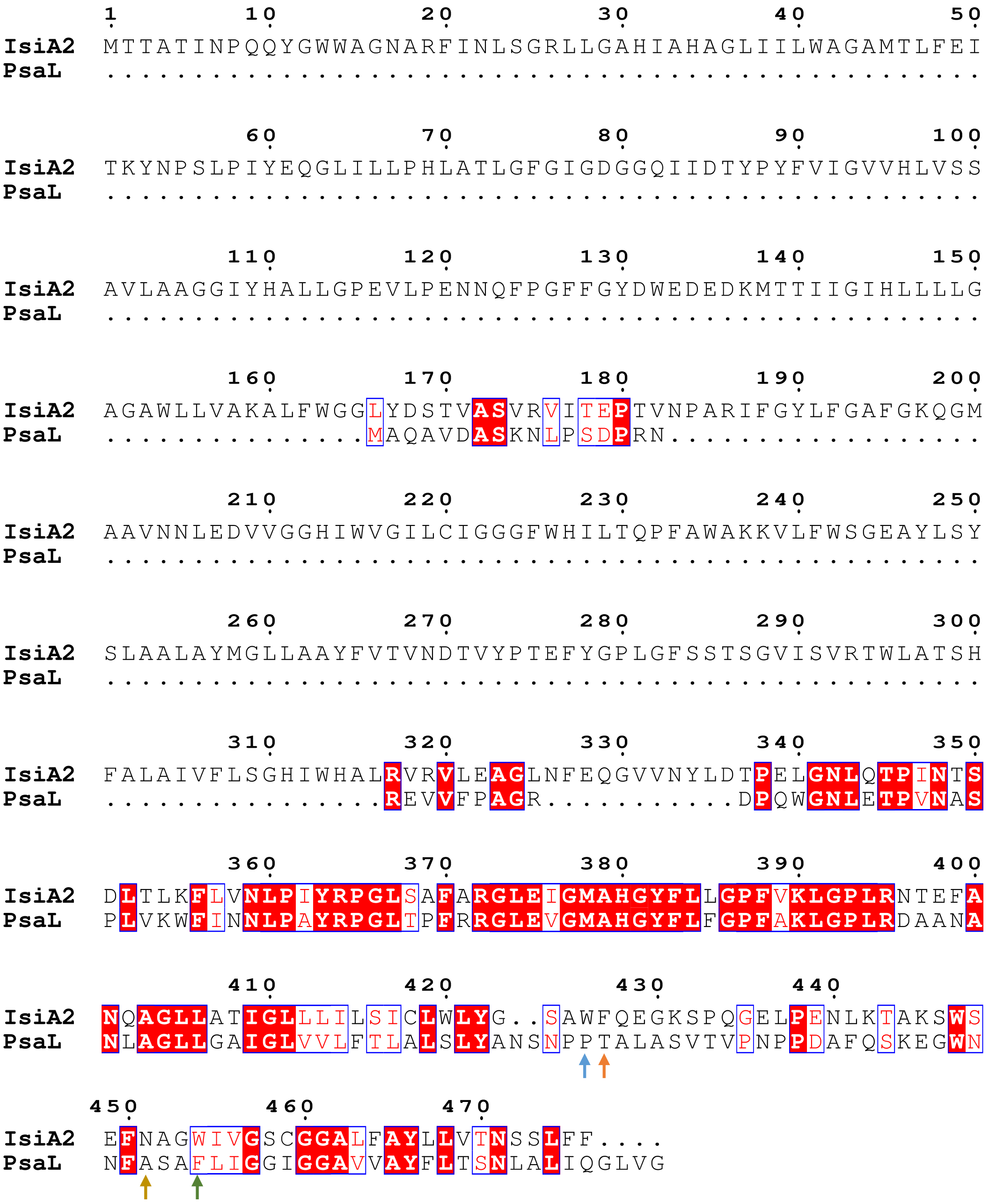

Supplementary Fig. 5. Sequence alignment of IsiA2 with PsaL of *Anabaena*.

Characteristic amino acids shown in Fig. 2c, d are labeled with arrows.

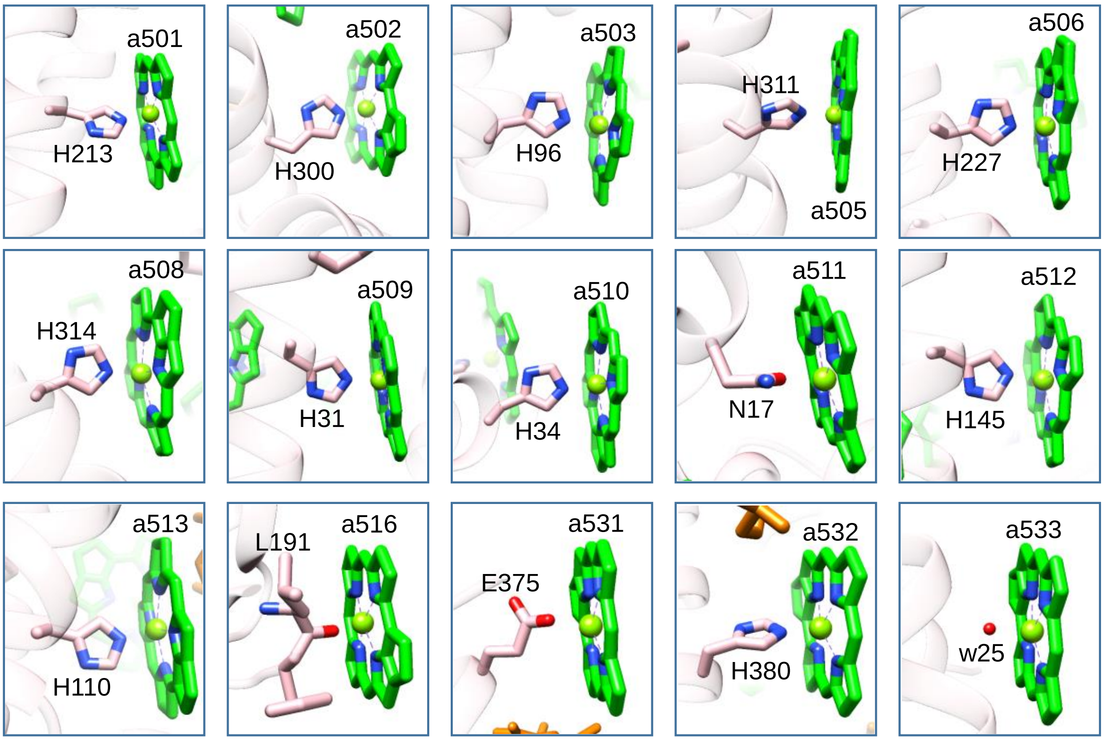

Supplementary Fig. 6. Chls and their ligands in IsiA2-1.

The rings of Chls are depicted and colored in green.

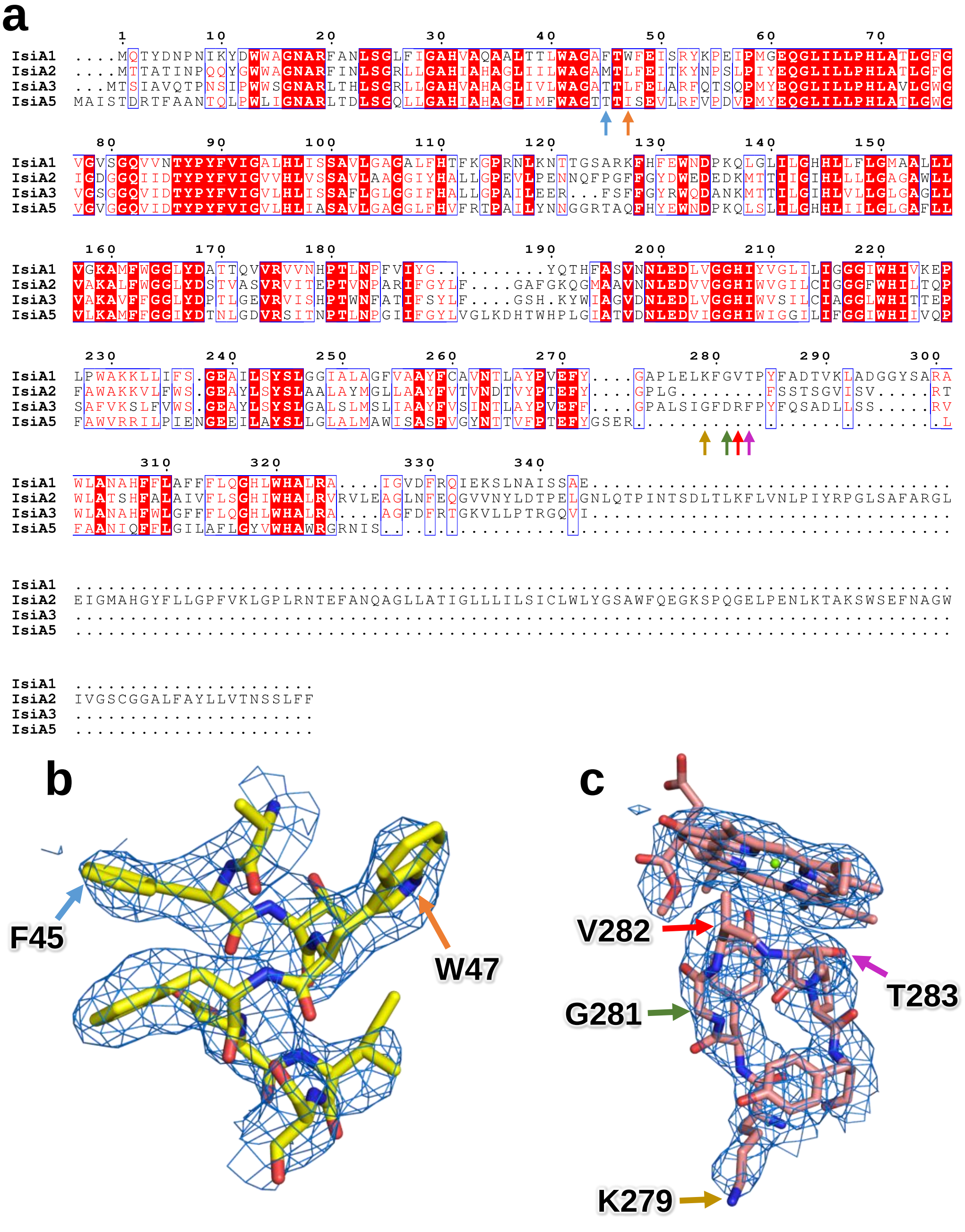

Supplementary Fig. 7. Characteristic amino-acid residues used for the identification of IsiA1.

a, Multiple sequence alignment (ClustalW and ESPript) of the four types of IsiAs in *Anabaena*. Unique residues are indicated by arrows with different colors, which were used for the identification of IsiA1. **b, c,** Characteristic maps and residues of IsiA1-4 (**b**) and IsiA1-5 (**c**). The maps are shown as meshes at 1 σ contour level, and the corresponding models are shown as sticks. The characteristic amino acids shown in panel **a** are labeled with the corresponding arrows.

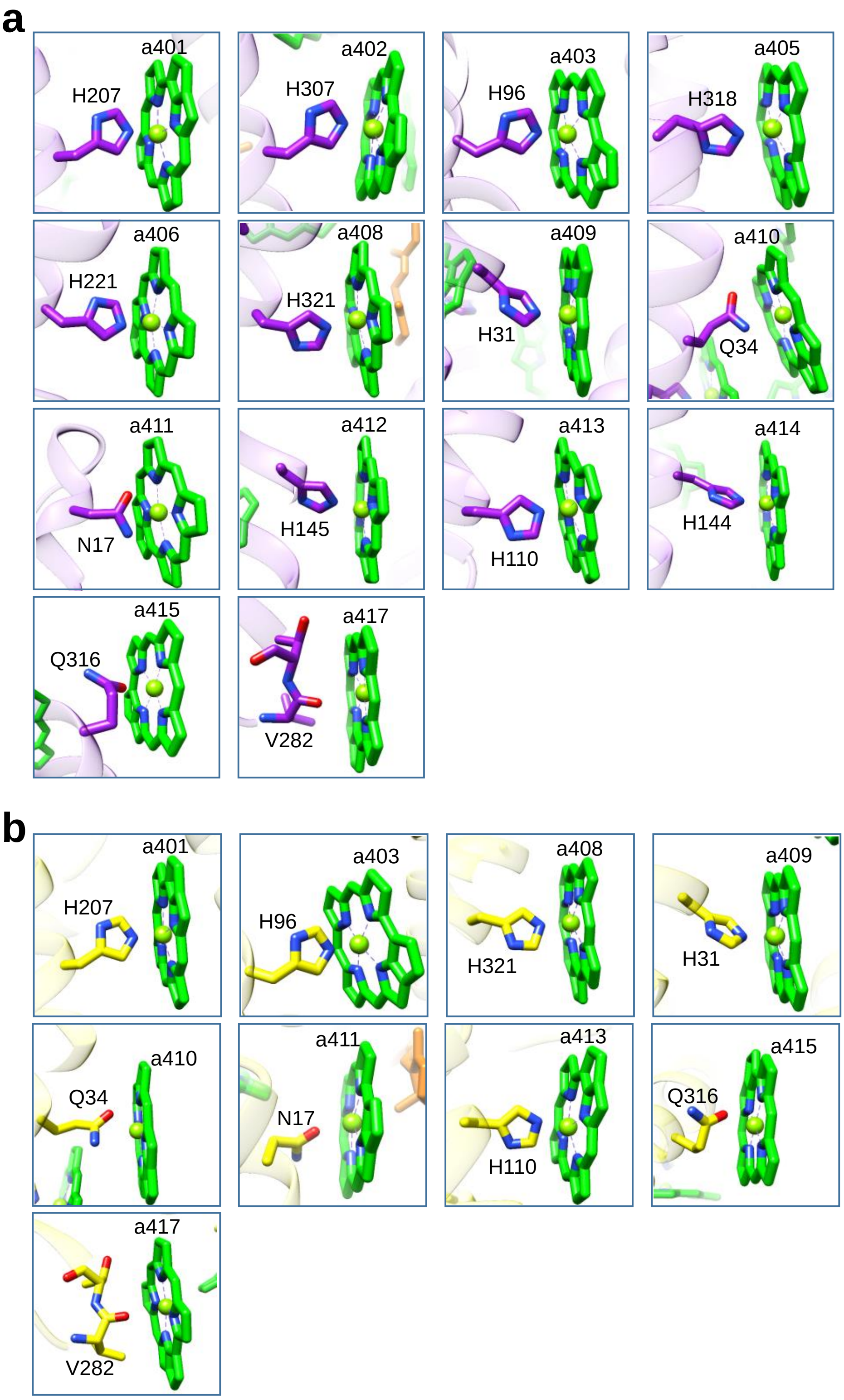

Supplementary Fig. 8. Chls and their ligands in IsiA1-5 and IsiA1-4.

The rings of Chls are depicted for IsiA1-5 (**a**) and IsiA1-4 (**b**) and colored in green.

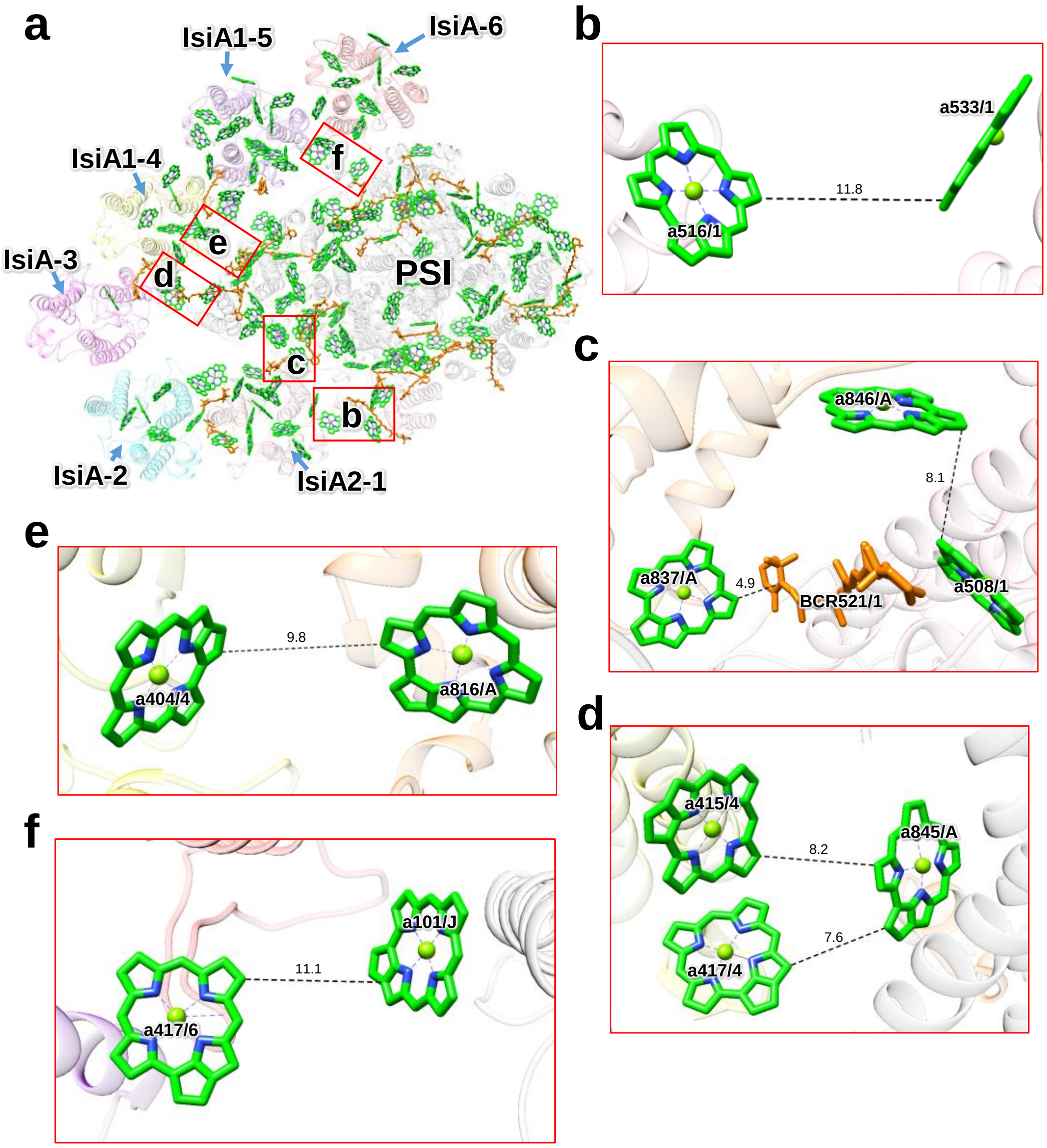

Supplementary Fig. 9. Pigment-pigment interactions between IsiAs and PSI.

a, The structure of PSI-IsiA viewed from the cytosolic side. Green squared areas are enlarged in panels **b**–**f**. **b,** Interaction between the N-terminal and C-terminal domains within IsiA2-1. **c**, Interactions between IsiA2-1 and PsaA. **d, e,** Interactions between IsiA1-4 and PsaA. **f**, Interaction between IsiA-6 and PsaJ. Only rings of the Chl molecules are depicted. Interactions are indicated by dashed lines, and the numbers are distances in Å. The letters represent the numbering of pigments in each subunit of IsiAs/PsaA/PsaJ; for example, a837/A means Chl *a* 837 in PsaA. BCR, *β*-carotene.

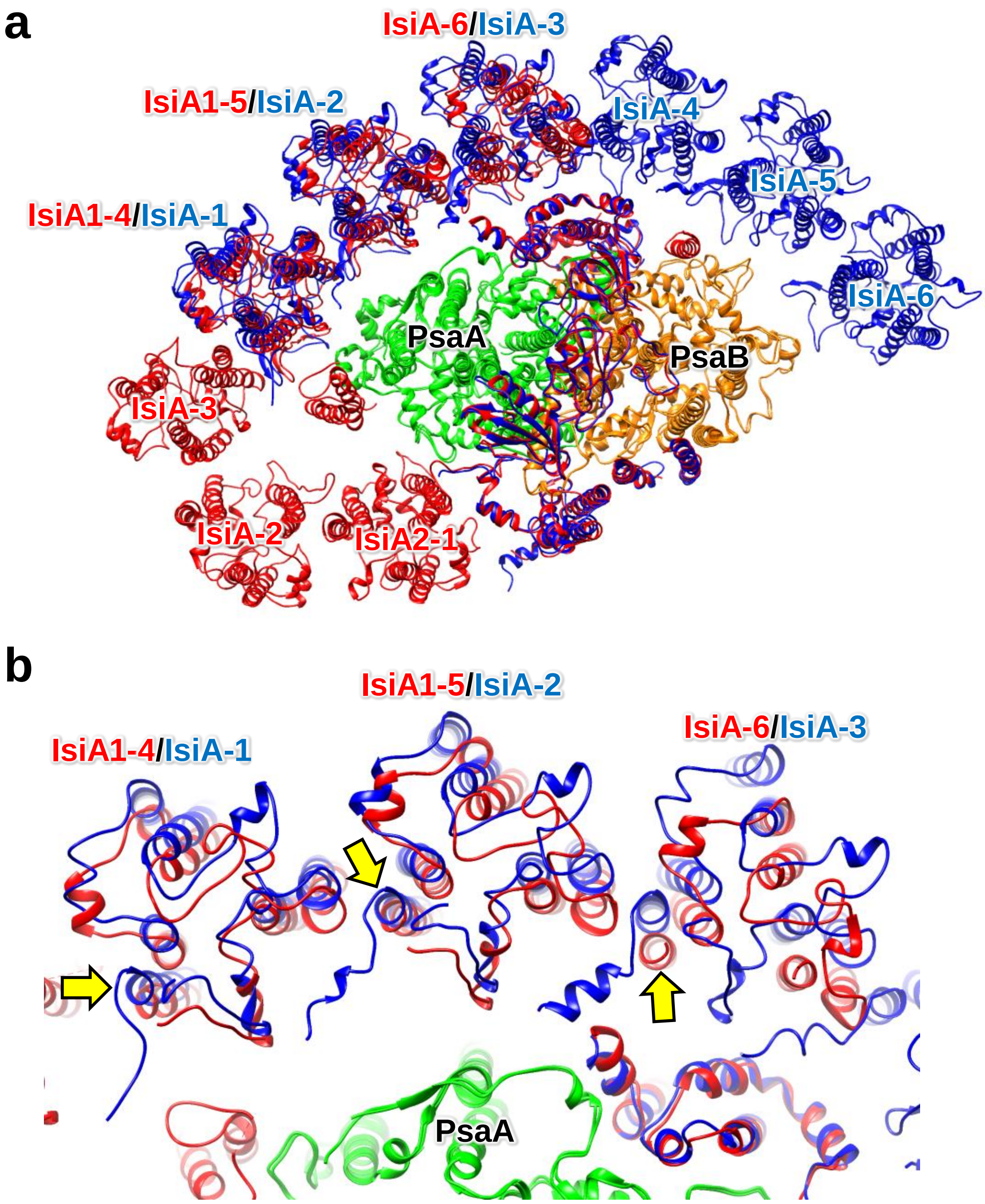
a
IsiA-6/IsiA-3
IsiA1-5/IsiA-2
IsiA-4
IsiA-5
IsiA1-4/IsiA-1
IsiA-6
PsaA
PsaB
IsiA-3
IsiA-2
IsiA2-1
b
IsiA1-5/IsiA-2
IsiA-6/IsiA-3
IsiA1-4/IsiA-1
PsaA

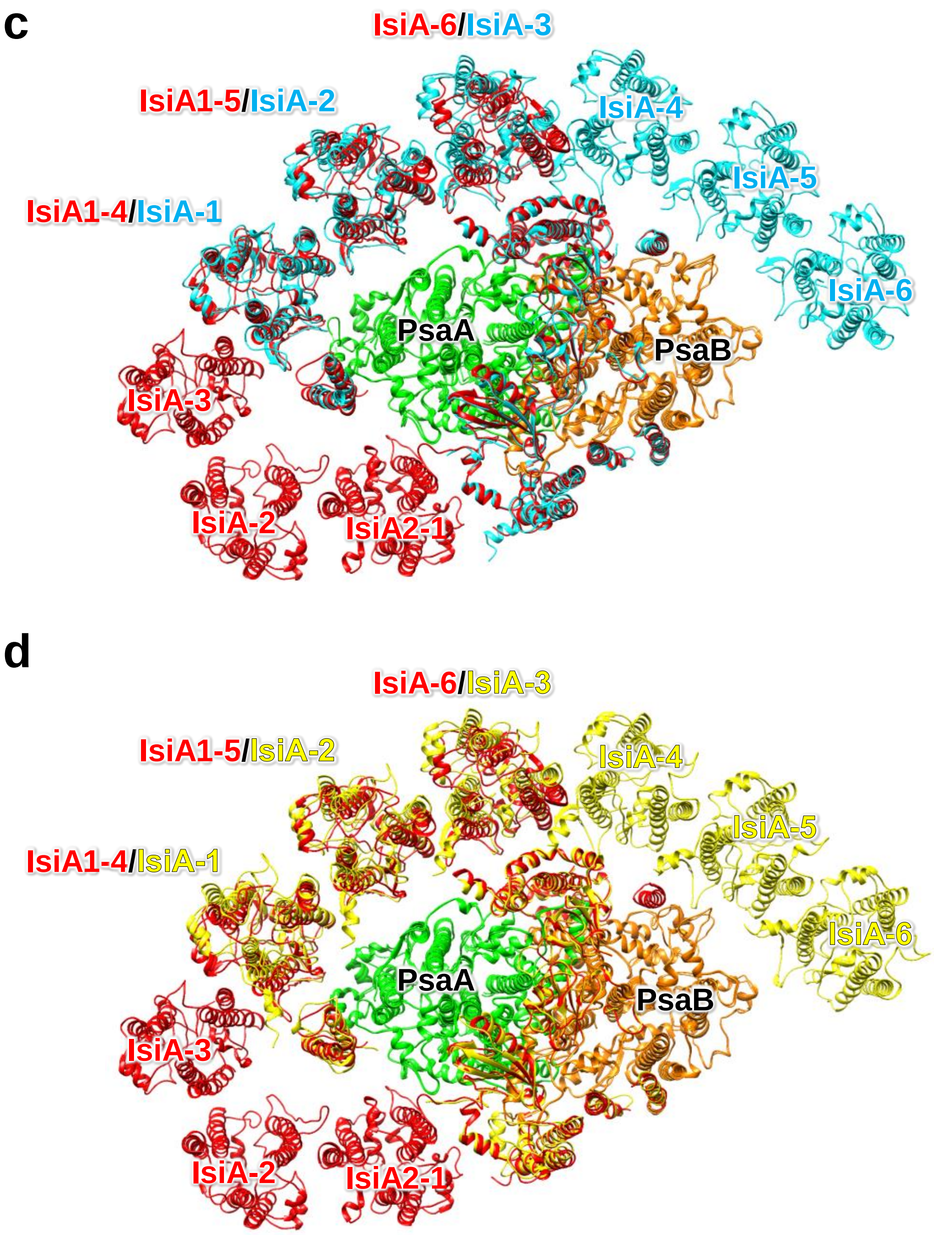

Supplementary Fig. 10. Structural comparisons of PSI-IsiA between *Anabaena* and other cyanobacteria.

The structures are viewed from the cytosolic side. Comparisons are made between *Anabaena* and *Synechocystis* sp. PCC 6803 (**a, b**), between *Anabaena* and *Thermosynechococcus vulcanus* NIES-2134 (**c**), and between *Anabaena* and *Synechococcus elongatus* PCC 7942 (**d**). The subunits of *Anabaena*, *Synechocystis*, *T. vulcanus*, and *Synechococcus* are colored red, blue, cyan, and yellow, respectively, with the colors of PsaA (green) and PsaB (orange) fixed in all the species. The six IsiA subunits in the *Synechocystis*, *T. vulcanus* and *Synechococcus* PSI-IsiA supercomplexes were named IsiA-1 to IsiA-6. **a, c, d,** overall structures; **b,** an expanded view at interfaces between IsiAs and PSI. The C-terminal loops of each IsiA are indicated by yellow arrows.

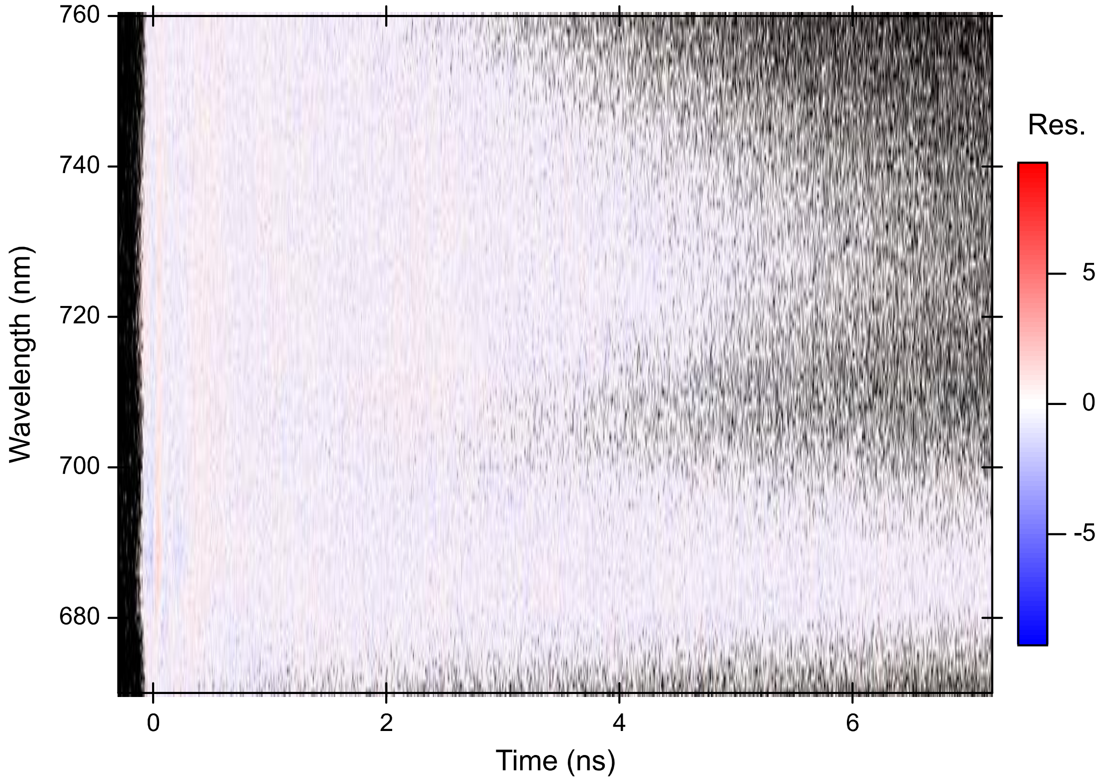

Supplementary Fig. 11. Residual map of the global analysis.

Residual (Res.) was defined as

$$Res. = \frac{I_m(t,\lambda) - I_c(t,\lambda)}{\sqrt{I_m(t,\lambda)}}.$$

Here, $I_m(t,\lambda)$ and $I_c(t,\lambda)$ are the measured and calculated data, respectively. Black dots indicate the points at which $I_m(t,\lambda)$ is 0.

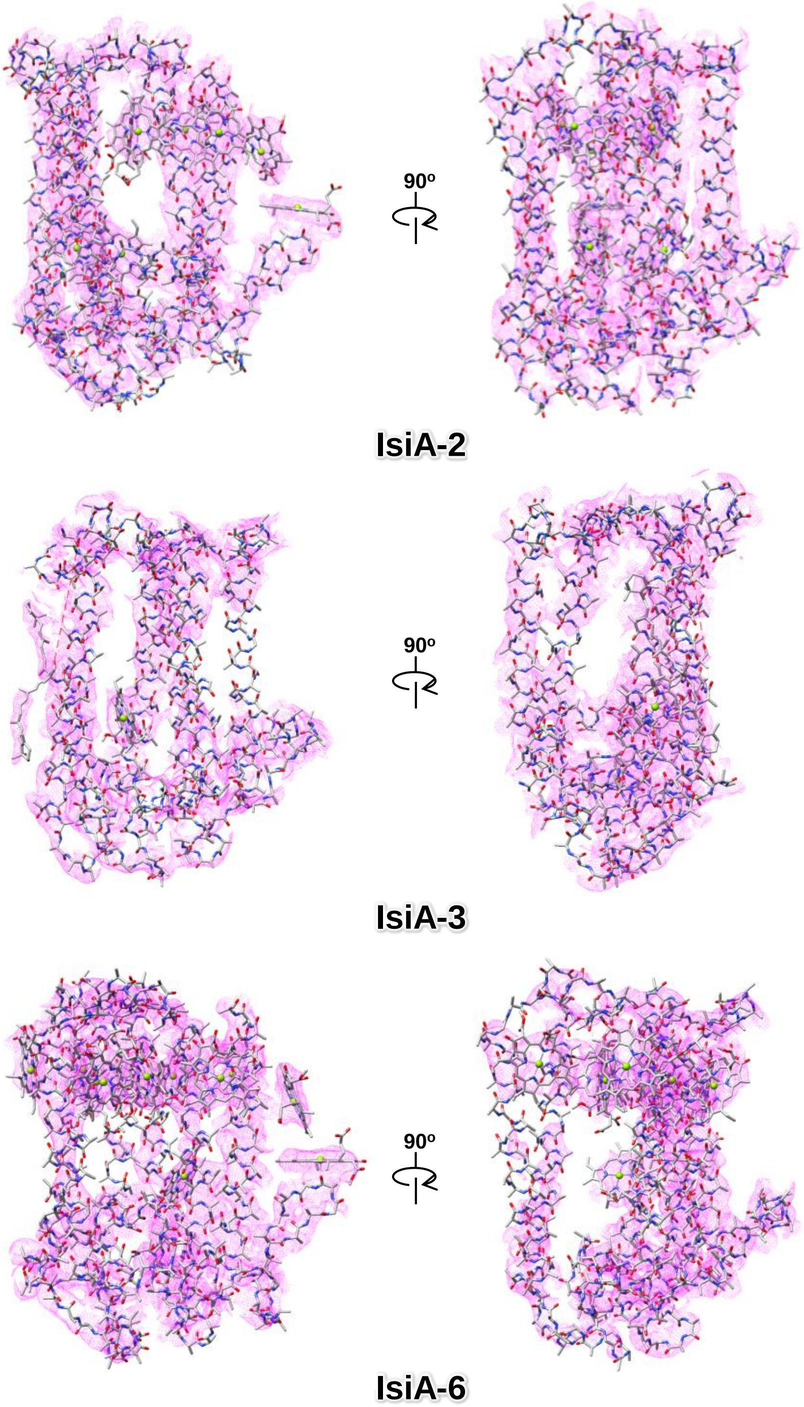

Supplementary Fig. 12. Evaluation of the maps and model for IsiA-2, IsiA-3, and IsiA-6. The densities for IsiA-2, IsiA-3, and IsiA-6 and their corresponding models are shown as magenta meshes and gray sticks, respectively, using the denoised map (see Methods).

Supplementary Table 1. Cryo-EM data collection and structural analysis statistics.

Complex	PSI-IsiA
PDB ID	7Y3F
EMDB ID	EMD-33593
Data collection and processing	
Magnification	100,000
Voltage (kV)	300
Electron exposure (e^-/Å)	40.8
Defocus range (μm)	−1.8 to −0.8
Pixel size (Å)	0.495
Symmetry imposed	C1
Final particle images (no.)	47,602
Map resolution (Å)	2.62
FSC threshold	0.143
Refinement	
Initial model used (PDB code)	Homology modeling
Model resolution (Å)	2.57
FSC threshold	0.5
Map sharpening B factor (Å^2)	−45.4
Model composition	
Non-hydrogen atoms	39,463
Protein	24,774
Ligand	14,663
Water	26
B factors (Å^2)	
Protein	97.7
Ligand	145.3
Water	44.3
R.m.s deviations	
Bond lengths (Å)	0.028
Bond angles (°)	2.86
Validation	
MolProbity score	1.78
Clashscore	7.96
Poor rotamers (%)	0.36
EMRinger score	4.53
Ramachandran plot	
Favored (%)	95.02
Allowed (%)	4.21
Disallowed (%)	0.77

Supplementary Table 2. Averaged *Q*-score in each subunit.

Subunit	Averaged *Q*-score	
	Postprocessed map	Denoised map
PsaA	0.80	0.78
PsaB	0.79	0.78
PsaC	0.81	0.77
PsaD	0.76	0.74
PsaE	0.73	0.72
PsaF	0.77	0.75
PsaI	0.78	0.76
PsaJ	0.78	0.76
Unknown	0.74	0.73
PsaM	0.76	0.74
PsaX	0.73	0.72
IsiA2-1	0.57	0.60
IsiA-2	0.24	0.34
IsiA-3	0.22	0.33
IsiA1-4	0.30	0.40
IsiA1-5	0.34	0.43
IsiA-6	0.25	0.40

Supplementary Table 3. Cofactors assigned in the current PSI-monomer-IsiA structure.

Protein	Chlorophyll	Carotenoid	Lipid	Others
**PsaA**	45 Chl *a*<br>1 Chl *a*'	7 BCR	2 LHG	1 [4Fe-4S] cluster,<br>1 phylloquinone
**PsaB**	41 Chl *a*	7 BCR	1 LMG<br>2 LHG	1 phylloquinone
**PsaC**	-	-	-	2 [4Fe-4S] cluster
**PsaD**	-	-	-	-
**PsaE**	-	-	-	-
**PsaF**	1 Chl *a*	1 BCR	-	-
**PsaI**	-	2 BCR	-	-
**PsaJ**	2 Chl *a*	3 BCR	-	-
**Unknown**	1 Chl *a*	-	-	-
**PsaM**	-	1 BCR	-	-
**PsaX**	1 Chl *a*	-	-	-
**IsiA2-1**	17 Chl *a*	5 BCR	-	-
**IsiA-2**	8 Chl *a*	-	-	-
**IsiA-3**	1 Chl *a*	1 BCR	-	-
**IsiA1-4**	10 Chl *a*	1 BCR	-	-
**IsiA1-5**	17 Chl *a*	1 BCR	-	-
**IsiA-6**	11 Chl *a*	-	-	-
**Total**	156	29	5	5

BCR, β-carotene; Chl *a*, chlorophyll *a*; LMG, distearoylmonogalactosyl diglyceride; LHG, dipalmitoylphosphatidyl glycerol.

Supplementary Table 4. Chls and their ligands in IsiA2-1, IsiA1-4, and IsiA1-5.

Protein	Chlorophyll/ligand
**IsiA2-1**	a501/H213, a502/H300, a503/H96, a504/-[1], a505/H311, a506/H227, a507/-[1], a508/H314, a509/H31, a510/H34, a511/N17, a512/H145, a513/H110, a516/L191, a531/E375, a532/H380, a533/w25[2]
**IsiA1-4**	a401/H207, a403/H96, a404/-[1], a408/H321, a409/H31, a410/Q34, a411/N17, a413/H110, a415/Q316, a417/V282
**IsiA1-5**	a401/H207, a402/H307, a403/H96, a404/-[1], a405/H318, a406/H221, a407/-[1], a408/H321, a409/H31, a410/Q34, a411/N17, a412/H145, a413/H110, a414/H144, a415/Q316, a416/-[1], a417/V282

[1]The ligands of these Chls may be water molecules that cannot be identified due to weak densities.
[2]Water molecule.

Supplementary Table 5. IsiA proteins identified in the *Anabaena* PSI-IsiA structure and their RMSD values with IsiA1-5.

Protein	Gene	RMSD (Å)/Aligned Cα atoms
**IsiA2-1**	*isiA2*	0.95/302
**IsiA-2**	unidentified	0.90/302
**IsiA-3**	unidentified	0.94/277
**IsiA1-4**	*isiA1*	0.56/326
**IsiA1-5**	*isiA1*	0.00/332
**IsiA-6**	unidentified	0.73/303

Supplementary Table 6. Primers used in this study.

Primer	Sequence (5′- -3′)
RTisiA1-F	CCCAAGCTGCTCTGACAAC
RTisiA1-R	AAAATAAGTCCTTGCTCACCCA
RTisiA2-F	GAGAGTGCGTGTCCTAGAGG
RTisiA2-R	CCCGGACGATAAATTGGCAG
RTisiA3-F	AACTAGCCCGTTTCCAGACA
RTisiA3-R	TAACCTGACCACCACTACCC
RTisiA5-F	GGGCAGGAACAACTACCATC
RTisiA5-R	ACTTGACCACCAACACCTACT
RTrrn16S-F2	GCAAGTCGAACGGTCTCTTC
RTrrn16S-R2	GGTATTAGCCACCGTTTCCA

Supplementary Table 7. Correspondence of numbering of pigments in each IsiA subunit described in the text with those in the PDB file.

IsiA2-1			IsiA-2	IsiA-3	IsiA1-4	IsiA1-5	IsiA-6
**Chls in the text**	**PDB No. (Chain ID)**	**Chls in the text**	**PDB No. (Chain ID)**	**PDB No. (Chain ID)**	**PDB No. (Chain ID)**	**PDB No. (Chain ID)**	**PDB No. (Chain ID)**
501	502 (1)	401	401 (2)	-	402 (4)	403 (5)	-
502	503 (1)	402	-	-	-	404 (5)	401 (6)
503	504 (1)	403	402 (2)	401 (3)	403 (4)	405 (5)	-
504	505 (1)	404	-	-	404 (4)	406 (5)	-
505	506 (1)	405	525 (1)*	-	-	407 (5)	421 (5)*
506	507 (1)	406	-	-	-	408 (5)	402 (6)
507	508 (1)	407	403 (2)	-	-	409 (5)	403 (6)
508	509 (1)	408	404 (2)	-	405 (4)	410 (5)	404 (6)
509	510 (1)	409	405 (2)	-	406 (4)	411 (5)	405 (6)
510	511 (1)	410	-	-	407 (4)	412 (5)	-
511	512 (1)	411	-	-	408 (4)	413 (5)	-
512	513 (1)	412	-	-	-	414 (5)	406 (6)
513	514 (1)	413	-	-	409 (4)	415 (5)	407 (6)
516	515 (1)	414	-	-	-	416 (5)	408 (6)
531	519 (1)	415	526 (1)*	-	410 (4)	417 (5)	422 (5)*
532	520 (1)	416	-	-	-	418 (5)	-
533	521 (1)	417	406 (2)	-	411 (4)	419 (5)	409 (6)
**Cars in the text**		**Cars in the text**					
521	516 (1)	421	-	-	-	420 (5)	-
522	517 (1)	424	-	401 (4)*	402 (5)*	-	-
524	518 (1)	-	-	-	-	-	-
534	522 (1)	-	-	-	-	-	-
535	523 (1)	-	-	-	-	-	-

*Chain in the adjacent unit.
