# Supplementary material for: Structure of a monomeric photosystem I core associated with iron-stress-induced-A proteins from Anabaena sp. PCC 7120
Source: Nat Commun. 2023 Feb 17;14:920. doi: 10.1038/s41467-023-36504-1 (PMC9938196; doi:10.1038/s41467-023-36504-1)
Supplement: Supplementary file 3 — Reporting Summary [file 41467_2023_36504_MOESM3_ESM.pdf]

nature portfolio

Corresponding author(s): Ryo Nagao

Last updated by author(s): Jan 12, 2023

Reporting Summary

Nature Portfolio wishes to improve the reproducibility of the work that we publish. This form provides structure for consistency and transparency in reporting. For further information on Nature Portfolio policies, see our Editorial Policies and the Editorial Policy Checklist.

Statistics

For all statistical analyses, confirm that the following items are present in the figure legend, table legend, main text, or Methods section.

n/a	Confirmed	
☐	☒	The exact sample size (*n*) for each experimental group/condition, given as a discrete number and unit of measurement
☐	☒	A statement on whether measurements were taken from distinct samples or whether the same sample was measured repeatedly
☒	☐	The statistical test(s) used AND whether they are one- or two-sided<br>*Only common tests should be described solely by name; describe more complex techniques in the Methods section.*
☒	☐	A description of all covariates tested
☒	☐	A description of any assumptions or corrections, such as tests of normality and adjustment for multiple comparisons
☐	☒	A full description of the statistical parameters including central tendency (e.g. means) or other basic estimates (e.g. regression coefficient) AND variation (e.g. standard deviation) or associated estimates of uncertainty (e.g. confidence intervals)
☒	☐	For null hypothesis testing, the test statistic (e.g. *F*, *t*, *r*) with confidence intervals, effect sizes, degrees of freedom and *P* value noted<br>*Give P values as exact values whenever suitable.*
☒	☐	For Bayesian analysis, information on the choice of priors and Markov chain Monte Carlo settings
☒	☐	For hierarchical and complex designs, identification of the appropriate level for tests and full reporting of outcomes
☒	☐	Estimates of effect sizes (e.g. Cohen's *d*, Pearson's *r*), indicating how they were calculated

Our web collection on statistics for biologists contains articles on many of the points above.

Software and code

Policy information about availability of computer code

Data collection	SerialEM 3-8, yoneoLocr 1
Data analysis	Relion 3.1, MotionCor2 1.4.2 , CTFFIND4 1.14, COOT 0.9.6, PHENIX 1.19.2-4158 (phenix.real_space_refine, phenix.molprobity, phenix.emringer), Pymol 2.5.0, UCSF Chimera 1.14, Servalcat 0.2.0, Q-score 1.6.2, JAFIS Tool version 1

For manuscripts utilizing custom algorithms or software that are central to the research but not yet described in published literature, software must be made available to editors and reviewers. We strongly encourage code deposition in a community repository (e.g. GitHub). See the Nature Portfolio guidelines for submitting code & software for further information.

Data

Policy information about availability of data

All manuscripts must include a data availability statement. This statement should provide the following information, where applicable:

- Accession codes, unique identifiers, or web links for publicly available datasets
- A description of any restrictions on data availability
- For clinical datasets or third party data, please ensure that the statement adheres to our policy

Atomic coordinate and cryo-EM maps for the reported structure have been deposited in the Protein Data Bank under an accession code 7Y3F [https://www.rcsb.org/structure/7Y3F] and in the Electron Microscopy Data Bank under an accession code EMD-33593 [https://www.ebi.ac.uk/emdb/EMD-33593]. Source data are provided with this paper. The data that support the findings of this study are available from the corresponding authors upon reasonable request.

Human research participants

Policy information about studies involving human research participants and Sex and Gender in Research.

Reporting on sex and gender	This paper dose not contain human research.
Population characteristics	This paper dose not contain human research.
Recruitment	This paper dose not contain human research.
Ethics oversight	This paper dose not contain human research.

Note that full information on the approval of the study protocol must also be provided in the manuscript.

Field-specific reporting

Please select the one below that is the best fit for your research. If you are not sure, read the appropriate sections before making your selection.

☒ Life sciences ☐ Behavioural & social sciences ☐ Ecological, evolutionary & environmental sciences

For a reference copy of the document with all sections, see nature.com/documents/nr-reporting-summary-flat.pdf

Life sciences study design

All studies must disclose on these points even when the disclosure is negative.

Sample size	No statistic method was used to predetermine the sample size. Amount of cryo-EM micrographs collected was based on the previous knowledge and experiences. The results obtained indicated that the images collected are sufficient to generate a whole density map at 2.62-Å resolution for the PSI-IsiA supercomplex.
Data exclusions	No data exclusion criteria was pre-established. 2D and 3D classification were used to obtain multiple classes. Only the particles in the classes that showed clear structural signals and intact structures were chosen, combined and used in the final reconstruction of cryo-EM map and refinement. Details are described in the flowchart of Supplementary Fig. S2 and Methods.
Replication	Multiple rounds of structural refinement have been performed and all resulted in the same density maps, although at different resolutions. Experiments concerning the purification and characterization of PSI-IsiA (Supplementary Fig. 1) have been repeated independently for more than 3 times, and all successfully reproduced. Three sets of TRF spectra were measured, and FDA spectra (Fig. 7) were analyzed by using data with the longest accumulation time.
Randomization	Randomization is not relevant to our study because we aimed to solve the structure of the specific protein supercomplex by cryo-electron microscopy. The density map was obtained by analyzing a final set of 47,602 particles, and the structure was analyzed with standard softwares which showed bond length and angle errors etc (Supplementary Table 1).
Blinding	Blinding is not relevant to our study because we aimed to solve the structure of the specific protein supercomplex.

Reporting for specific materials, systems and methods

We require information from authors about some types of materials, experimental systems and methods used in many studies. Here, indicate whether each material, system or method listed is relevant to your study. If you are not sure if a list item applies to your research, read the appropriate section before selecting a response.

Materials & experimental systems

n/a	Involved in the study	
☒	☐	Antibodies
☒	☐	Eukaryotic cell lines
☒	☐	Palaeontology and archaeology
☒	☐	Animals and other organisms
☒	☐	Clinical data
☒	☐	Dual use research of concern

Methods

n/a	Involved in the study	
☒	☐	ChIP-seq
☒	☐	Flow cytometry
☒	☐	MRI-based neuroimaging
